# WEARABLES REVEAL A GAP BETWEEN GAIT PERFORMANCE IN THE LAB AND DURING 24/7 MONITORING IN OLDER ADULTS

**DOI:** 10.1093/geroni/igz038.1218

**Published:** 2019-11-08

**Authors:** Inbar Hillel, Laura Avanzino, Andrea Cereatti, Marcel Olde Rikkert, Silvia Del Din, Pieter Ginis, Anat Mirelman, Jeffrey M Hausdorff

**Affiliations:** 1 Center for the study of Movement, Cognition and Mobility, Neurological Institute, Tel Aviv Sourasky Medical Center, Israel, Tel Aviv, Israel; 2 IRCCS San Martino Teaching Hospital, Genoa, Italy., Genoa, Italy; 3 Department of Biomedical Sciences, Bioengineering unit, University of Sassari, Italy, Sassari, Italy; 4 Department of Geriatric Medicine, Donders Centre for Medical Neuroscience, Radboudumc Alzheimer Center, Radboud university medical center, Nijmegen, The Netherlands, Nijmegen, Netherlands; 5 Institute of Neuroscience, Newcastle University Institute for Ageing, Clinical Ageing Research Unit, Campus for Ageing and Vitality, Newcastle University, Newcastle upon Tyne, UK, Newcastle upon Tyne, United Kingdom; 6 Neuromotor Rehabilitation Research Group, Department of Rehabilitation Sciences, KU Leuven, Belgium, Leuven, Belgium

## Abstract

We compared in-lab usual-walking (UW) and dual-task walking (DTW) to daily-living measures of gait obtained during 24/7 monitoring. In-lab gait features (e.g., gait speed, step and stride regularity) derived from UW and DTW were compared to the same gait features during daily-living in 150 elderly fallers (age: 76.5±6.3 years, 37.6% men). Features were extracted from a lower-back accelerometer. In daily-living setting, subjects wore the device for one week and pre-processing detected 30-second walking bouts. A histogram of all walking bouts was determined for each walking feature for each subject, then each subject’s typical, worst and best values were determined. Statistics of reliability were assessed using ICC and Bland-Altman. As expected, in-lab gait speed, step regularity, and stride regularity were worse during DTW, compared to UW. Gait speed, step regularity, and stride regularity during UW were significantly higher (i.e., better) from the typical daily-living values (p<0.0001) and different (p<0.000) from the worst and best values. DTW values tended to be similar to typical daily-living values (p=0.205, p=0.053, p=0.013 respectively). ICC assessment and Bland-Altman plots indicated that in-lab values do not reliably reflect the daily-walking values. Gait values during relatively long daily-living walking bouts are more similar to the corresponding values obtained in the lab during DTW, as compared to UW. Still, gait performance during most daily-living walking bouts are worse than that measured in-lab and do not reliably reflect each other. That is, an older adult’s typical daily-living gait cannot be estimated by simply measuring walking in a structured, laboratory setting.

